# A double‐blind, randomized, multicenter phase 3 study of palonosetron vs granisetron combined with dexamethasone and fosaprepitant to prevent chemotherapy‐induced nausea and vomiting in patients with breast cancer receiving anthracycline and cyclophosphamide

**DOI:** 10.1002/cam4.2979

**Published:** 2020-03-13

**Authors:** Koji Matsumoto, Masato Takahashi, Kazuhiko Sato, Akihiko Osaki, Toshimi Takano, Yoichi Naito, Kazuo Matsuura, Kenjiro Aogi, Kimiko Fujiwara, Kenji Tamura, Motoi Baba, Shinya Tokunaga, Gen Hirano, Shigeru Imoto, Chieko Miyazaki, Kazuhiro Yanagihara, Chiyo K. Imamura, Yasutaka Chiba, Toshiaki Saeki

**Affiliations:** ^1^ Hyogo Cancer Center Hyogo Japan; ^2^ NHO Hokkaido Cancer Center Hokkaido Japan; ^3^ Tokyo‐West Tokushukai Hospital Tokyo Japan; ^4^ Saitama Medical University Saitama Japan; ^5^ Toranomon Hospital Tokyo Japan; ^6^ National Cancer Center Hospital East Kashiwa Japan; ^7^ Hiroshima Prefectural Hospital Hiroshima Japan; ^8^ Shikoku Cancer Center Ehime Japan; ^9^ Kindai University Hospital Osaka Japan; ^10^ National Cancer Center Hospital Tokyo Japan; ^11^ Hokkaido University Hokkaido Japan; ^12^ Osaka City General Hospital Osaka Japan; ^13^ Kyushu Hospital Fukuoka Japan; ^14^ Kyorin University Tokyo Japan; ^15^ Jichi Medical University Tochigi Japan; ^16^ Kansai Electric Power Hospital Osaka Japan; ^17^ Advanced Cancer Translational Research Institute Showa University Tokyo Japan

**Keywords:** AC regimen, CINV, fosaprepitant, granisetron, palonosetron

## Abstract

**Purpose:**

To investigate whether palonosetron is better than granisetron in preventing chemotherapy‐induced nausea and vomiting (CINV) in a three‐drug combination with dexamethasone and fosaprepitant (Fos) in patients with breast cancer who are placed on anthracycline and cyclophosphamide (AC‐based regimen).

**Patients and Methods:**

Chemo‐naive women with primary breast cancer were randomly administered either palonosetron 0.75 mg (day 1) or granisetron 1 mg (day 1) combined with dexamethasone (12 mg at day 1, 8 mg at day 2 and day 3) and Fos 150 mg (day 1) before receiving AC‐based regimen in a double‐blind study. The primary endpoint was the complete response (CR) rate of emesis in cycle 1 in the delayed phase. This was defined as neither vomiting nor rescue drug usage for emesis at >24‐120 hours after chemotherapy. Secondary endpoints were the CR in the acute/overall phase (0‐24/0‐120 hours, respectively, after chemotherapy), no nausea and vomiting, Patient‐Reported Outcomes version of the Common Terminology Criteria for Adverse Events (PRO‐CTCAE), and safety.

**Results:**

From December 2012 to October 2014, 326 patients were treated and evaluated (164/162 evaluable patients in granisetron/palonosetron arm, respectively). The CR during the delayed phase was 60.4% in the granisetron regimen and 62.3% in the palonosetron regimen. The CR during acute phase (73.2% vs 75.9%, respectively) and the CR during overall phase (54.9% in both regimens) were very identical. A significantly higher number of patients in the palonosetron arm were free from nausea during the delayed phase (28% vs 40.1%; *P* = .029). Adverse events were also identical, although infusion site reactions (ISR) were higher (20.3%‐23.3%) than preceding studies in both regimens.

**Conclusion:**

In combination with dexamethasone and Fos, this study suggests that palonosetron is not better than granisetron in chemo‐naive patients with primary breast cancer receiving AC‐based regimen. Administration of Fos in peripheral veins after AC‐based regimen increased ISR.

## Graphical abstract

A randomized phase 3 trial compared palonosetron with granisetron as combination therapy with dexamethasone and fosaprepitant for chemotherapy‐induced nausea and vomiting prevention in breast cancer patients receiving anthracycline and cyclophosphamide. Although palonosetron was better than granisetron in terms of control of nausea in the delayed phase, the primary endpoint, CR in the delayed phase, was not statistically significant (62.3% vs 60.4%).

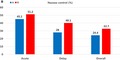

## 1 INTRODUCTION

Breast cancer is the most common type of cancer affecting women in Japan. Its standard perioperative chemotherapy regimen comprises a combination of anthracycline and cyclophosphamide regimen (AC‐based regimen) such as doxorubicin + cyclophosphamide (AC), epirubicin + cyclophosphamide (EC), or 5‐fluorouracil (5FU) + AC (FAC) or EC (FEC). All these combinations are associated with a high risk of chemotherapy‐induced nausea and vomiting (CINV), the most common adverse event for patients with breast cancer.

The use of effective antiemetics, such as steroids, serotonin receptor antagonists (5‐HT3 RAs), and neurokinin 1 (NK‐1) inhibitors (NK‐1 RAs), drastically improves CINV. In this regard, a three‐drug combination has been recommended for patients with breast cancer who are receiving AC‐based regimen on the basis of three major clinical guidelines: the American Society of Clinical Oncology (ASCO) guidelines,^1^ the National Comprehensive Cancer Network (NCCN) Clinical Practice Guidelines in Oncology,^2^ and the Multinational Association of Supportive Care in Cancer (MASCC).^3^

Palonosetron, a second‐generation 5‐HT3 RA, has a longer half‐life than other first‐generation 5‐HT3 RAs. The PROTECT trial was the first trial that compared palonosetron to granisetron combined with dexamethasone for patients receiving highly emetogenic chemotherapy (HEC) such as cisplatin (CDDP) or AC‐based regimen. In that trial, palonosetron was better than granisetron as the primary endpoint, which is complete response (CR: no vomiting and no rescue usage) in delayed phase (>24‐120 hours (h) after the chemotherapy) for patients receiving CDDP or AC‐based regimen combined with dexamethasone.^4^ In subgroup analysis for patients receiving AC‐based regimen, the CR during delayed phase and the CR during acute phase (0‐24 hours post chemotherapy) was 50% vs 61.1% and 64.8% vs 69% in granisetron and palonosetron, respectively. One limitation of the PROTECT study is that it did not use NK‐1 RAs. A systematic review and meta‐analysis revealed that palonosetron is better than first‐generation 5‐HT3 RAs, although none of the eight trials included in the meta‐analysis used NK‐1 RAs.^5^ Therefore, it remains unknown whether palonosetron is better than first‐generation 5‐HT3 RAs when combined with both dexamethasone and NK‐1 RAs as stated in the ASCO guidelines.^6^

Fosaprepitant dimeglumine (Fos), a water‐soluble, phosphorylated analog of aprepitant, is rapidly converted to aprepitant after intravenous (IV) administration. The EASE study showed that a triple‐antiemetic regimen containing a single dose of IV Fos is noninferior to a triple‐antiemetic regimen with 3 days of oral administration of aprepitant.^7^

This study seeks to investigate whether a three‐drug combination of palonosetron with dexamethasone and Fos is better than granisetron + dexamethasone + Fos in preventing CINV in patients with breast cancer receiving AC‐based regimen.

## 2 PATIENTS AND METHODS

### 2.1 Study design and treatment

The West Japan Oncology Group (WJOG) 6811B study (UMIN000008897) is a double‐blind, active‐controlled, multicenter phase 3 trial that evaluates the efficacy and safety of palonosetron or granisetron combined with dexamethasone and Fos for chemo‐naive patients with breast cancer receiving AC‐based regimen in cycle one. Patients were randomly assigned to palonosetron or granisetron treatment groups after stratification using minimization method. Stratification factors included institution, age (<55 years or ≥55 years), and the chemotherapy regimen (AC/EC/FAC/FEC). After stratification, the patients were randomly assigned to palonosetron or granisetron treatment. It was ensured that treatment assignments were made by personnel that were not involved in the study at each institution. The dose of the administered palonosetron was 0.75 mg, not 0.25 mg. This dose is the approved dose in Japan based on randomized studies previously conducted in Japan.^8^, ^9^ A subsequent systematic review reported that the doses of 0.25 and 0.75 mg achieved same therapeutic efficacy.^5^ The dose of administered granisetron in the study was 1 mg.

The inclusion and exclusion criteria for the study were as follows: women aged ≥20 years with invasive breast cancer, an Eastern Cooperative Oncology Group (ECOG) performance status of 0‐2, adequate organ function, and no history of chemotherapy. Antiemetic medication in the past 72 hours before enrollment was not allowed. All patients received dexamethasone (12 mg on day 1, 8 mg on day 2 and 3) and Fos 150 mg at day 1. To minimize phlebitis, Fos was diluted with ≥250 mL of normal saline and infused for ≥30 min.

The study protocol was approved by the institutional review board of each institution. All participants provided written informed consent before participating in the study.

### 2.2 Assessments

The primary endpoint was the complete response (CR) rate of emesis in the delayed phase and was defined as the percentage of patients without vomiting or rescue drug usage for emesis during >24‐120 hours after chemotherapy. Secondary endpoints were the CR rate of emesis in the acute phase, the CR rate of emesis in the overall phase (0‐120 hours after chemotherapy); the CR rates of nausea or vomiting during acute, delayed, and overall phases; patient‐reported outcomes (PRO); and safety (using Common Terminology Criteria for Adverse Events [CTCAE] ver. 4). Treating physicians answered the causality of each adverse events (multiple choice allowed). Severe adverse events (SAEs) were defined as adverse events related to hospitalization, prolongation of hospitalization, or death. All patients were asked to maintain a diary to report episodes of nausea, usage of rescue drugs, vomiting, adverse events, and PROs. PROs include two questions regarding the frequency and severity of nausea.

### 2.3 Statistical analyses

On the basis of previous studies,^10^, ^11^, ^12^, ^13^, ^14^ we expected a 65% CR during delayed phase in regimen A (granisetron) and an 80% CR during delayed phase in regimen B (palonosetron). To perform a chi‐square test with continuity correction at two‐sided significance level of .05 and a power of 0.80, we determined that 302 patients will be needed in the PPS. Assuming that 8% of the patients were ineligible or lost to follow‐up, we planned the study sample size as 330.

The proportion of the primary endpoint, the CR during delayed phase, was obtained for each regimen. Chi‐square test with the continuity correction, where the significance level was .05 (two‐sided) was used for comparison. We performed explanatory subgroup analyses of some background factors and multivariable logistic regression analysis with age, chemotherapy regimen, body mass index (BMI), drinking habit, motion sickness, and morning sickness as explanatory variables, in addition to the regimen. We performed similar analyses for the secondary endpoints.

## 3 RESULTS

### 3.1 Study conducts

**FIGURE 1:**
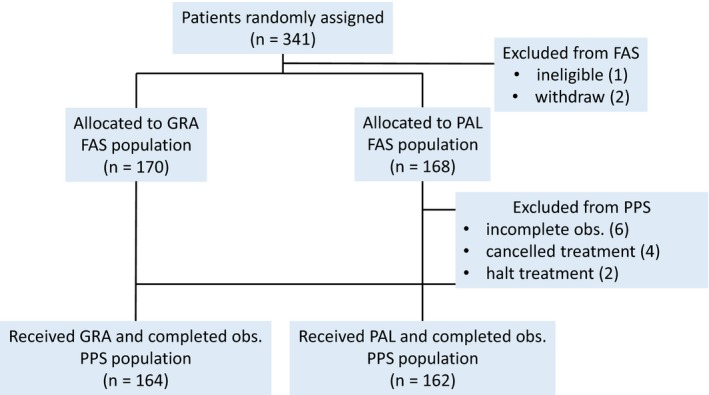
CONSORT flow diagram. Abbreviations: FAS, full analysis set; GRA, granisetronnisetron; PAL, palonosetron; PPS, per‐protocol set; obs, observation

From December 2012 to October 2014, 341 patients were randomized. Three patients were ineligible or withdrew their consent before the start of the treatment. The three were all removed from the FAS dataset. Four patients did not start the treatment protocol (delaying more than 2 weeks due to personal events or laboratory findings), two patients did not complete the treatment protocol (due to allergic reaction by anticancer agents), and six patients did not complete the protocol specified for the follow‐up. After adjusting for all these factors, the PPS dataset includes 326 patients. The CONSORT flaw is shown in Figure 1.

### 3.2 Patient characteristics

The patient characteristics are summarized in Table 1.

**TABLE 1:** Patient characteristics

	GRANISETRON n = 164	PALONOSETRON n* *= 162
Age (years), median (range)	54 (27‐82)	54 (30‐74)
<55, n (%)	87 (53%)	83 (51.2%)
≥55, n (%)	77 (47%)	79 (48.8%)
Height (cm), median (range)	156 (139‐174)	156 (144‐167)
Weight (kg), median (range)	54.5 (35.2‐91.8)	55.9 (36.3‐86.0)
BMI (kg/m^2^), median (range)	22.2 (16.0‐36.7)	23.1 (15.8‐35.0)
<25, n (%)	126 (76.8%)	113 (69.8%)
≥25, n (%)	38 (23.2%)	49 (30.2%)
ECOG performance status, n (%)		
0	163 (99.4%)	161 (99.4%)
1	1 (0.6%)	1 (0.6%)
2	0 (0%)	0 (0%)
Chemotherapy regimen, n (%)		
AC	21 (12.8%)	19 (11.7%)
EC	47 (28.7%)	49 (30.2%)
FAC	0 (0.0%)	0 (0.0%)
FEC	96 (58.5%)	94 (58.0%)
History of drinking, n (%)		
Yes	41 (25.0%)	50 (30.9%)
No	121 (73.8%)	110 (67.9%)
Unknown	2 (1.2%)	2 (1.2%)
Motion sickness, n (%)		
Yes	40 (24.4%)	42 (25.9%)
No	117 (71.3%)	114 (70.4%)
Unknown	7 (4.3%)	6 (3.7%)
Morning sickness, or nulliparity, n (%)		
Yes	89 (54.3%)	81 (50.0%)
No	51 (31.1%)	51 (31.5%)
Nulliparity	17 (10.4%)	25 (15.4%)
Unknown	7 (4.3%)	5 (3.1%)
Treatment setting, n (%)		
Adjuvant	81 (49.4%)	92 (56.8%)
Neoadjuvant	83 (50.6%)	70 (43.2%)

Abbreviations: AC, doxorubicin + cyclophosphamide; BMI, body mass index; EC, epirubicin + cyclophosphamide; ECOG, Eastern Cooperative Oncology Group;FAC, 5‐fluorouracil + doxorubicin + cyclophosphamide; FEC, 5‐fluorouracil + epirubicin + cyclophosphamide; GRANISETRON, granisetron; PALONOSETRON, palonosetron.

Almost half of the patients were young (<55 years), with a quarter being overweight (BMI > 25 kg/m^2^). In addition, 88% of the patients were on epirubicin, 30% had a drinking habit, 25% had motion sickness, and almost half had a history of morning sickness. These patient characteristics were well balanced between regimens A and B.

### 3.3 Fos infusion time

In this study, 83.7%, 6.7%, 2.1%, and 1.8% of patients received Fos for 30‐40, 40‐50, 60, and >60 minutes, respectively.

### 3.4 Efficacy

#### 3.4.1 Primary endpoint and subgroup analysis

**FIGURE 2:**
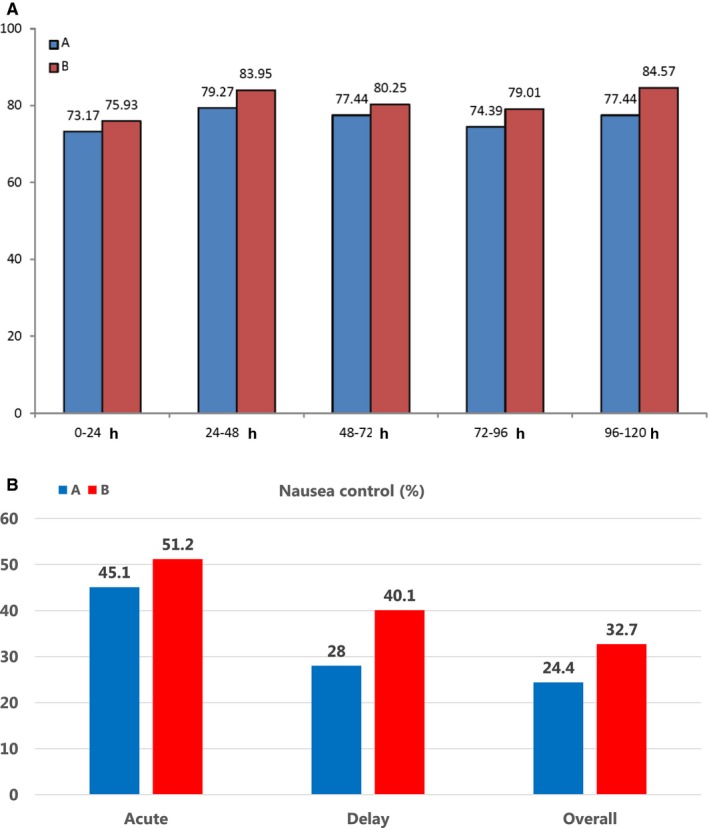
(A) Complete response in 120 hours after chemotherapy. Abbreviations: hrs, hours; A, granisetron; B, palonosetron. (B) Nausea control during each phase after chemotherapy. Abbreviations: Acute, 0‐24 hours after chemotherapy; Delay, >24‐120 hours after chemotherapy: Overall, 0‐120 hours after chemotherapy

For the primary endpoint, the CR during the delayed phase was 60.4% (99/164) vs 62.3% (101/162) in patients receiving regimen A and regimen B, respectively (*P* = .8). Although the CR rate in every 24‐hours period was better in regimen B (Figure 2A) than in regimen A, the CR during the overall phase was identical in both regimens [54.9% (90/164) vs 54.9% (89/162) (*P* = 1.0)].

Multivariable logistic regression analysis (Table 2) revealed the following: First, a history of motion sickness was a statistically significant risk factor for the CR during acute phase (OR = 0.57; 95% CI 0.32‐1.01; *P* = .053), the CR during delayed phase (OR = 0.51; 95% CI 0.30‐0.88; *P* = .015), and the CR during overall phase (OR = 0.49; 95% CI 0.29‐0.84; *P* = .009).

**TABLE 2:** Logistic regression analysis of predictive factors for CINV

		CR during delayed phase		CR during acute phase		CR during overall phase	
		OR	*P*	OR	*P*	OR	*P*
Age	>55/<	0.87	0.585	1.81	0.037	1.23	0.402
Rx	AC/FEC	0.3	0.005	0.48	0.107	0.32	0.008
	EC/FEC	1.05	0.885	0.81	0.549	0.75	0.342
BMI	>25/<	0.7	0.189	1.28	0.431	0.67	0.14
Drinking	Yes/no	0.77	0.333	0.88	0.653	0.75	0.273
Motion	Yes/no	0.51	0.015	0.57	0.053	0.49	0.009
Morning	Yes/no	0.69	0.176	0.71	0.271	0.68	0.153

Abbreviations: AC, doxorubicin + cyclophosphamide; BMI, body mass index; CINV, chemotherapy‐induced nausea and vomiting; drinking, drinking habit; EC, epirubicin + cyclophosphamide; FEC, 5‐fluorouracil + epirubicin + cyclophosphamide; morning, history of morning sickness; motion, history of motion sickness; OR, odds ratio; Rx, regimen.

Second, AC as a regimen was also a statistically significant risk factor for the CR during delay phase (OR = 0.30; 95% CI 0.13‐0.70; *P* = .005) and the CR during overall phase (OR = 0.32; 95% CI 0.14‐0.74; *P* = .008). Third, age was a statistically significant risk factor only for the CR during acute phase (OR = 1.81; 95% CI 1.03‐3.16; *P* = .037). Considering these risk factors, none of the planned subgroup analyses showed significant differences between regimens A and B. The BMI, drinking habit, and morning sickness were not risk factors for the CR during acute, delayed, and overall phase.

#### 3.4.2 Secondary endpoints

The percentage of patients without vomiting during the acute, delayed, and overall phases after the administration of regimens A and B was 93.9% (154/164) vs 95.7% (155/162) (*P* = .636), 82.3% (135/164) vs 89.5% (145/162) (*P* = .088), and 79.9%(131/164) vs 85.8% (139/162) (*P* = .203), respectively. The percentage of patients without nausea during the acute, delayed, and overall phases after administration of regimens A and B was 45.1% (74/164) vs 51.2% (83/162) (*P* = .32), 28.0% (46/164) vs 40.1% (65/162) (*P* = .029), and 24.4% (40/164) vs 32.7% (53/162) (*P* = .123), respectively (Figure 2B). Regarding the PROs, the frequency of nausea was lower after the administration of regimen B compared to regimen A (*P* = .014).

#### 3.4.3 Safety

Three patients experienced SAEs during the study period: delirium after febrile neutropenia (regimen B), acute pancreatitis (regimen A), and febrile neutropenia (regimen B). None of the SAEs was considered to be related to the use of antiemetics. The nonhematologic toxicities are summarized in Table 3. Adverse events related to antiemetics include constipation, headache, and infusion site reaction (ISR). Regarding ISR, among 72 patients experiencing ISR (38 patients in regimen A and 34 patients in regimen B), the physicians judged 68 (94.4%) ISRs as related to chemotherapy and 40 (55.5%) ISRs as related to antiemetics (Fos). Most of these adverse events were grade 1 or 2. We observed no differences between regimens A and B.

**TABLE 3:** Nonhematologic adverse events experienced by two or more patients

	Granisetron (%)				Palonosetron (%)			
	G1	G2	G3	Any G	G1	G2	G3	Any G
Nausea	47	9	0.6	56.8	43	8	0	51.4
Vomiting	10.8	2.4	0.6	13.7	10.8	1.2	0	11.9
Constipation	26.9	0.6	0	27.5	31.7	2.4	0	34
Headache	12	0	0	12	13.2	0	0	13.2
ISR	21.6	1.2	0.6	23.3	18	2.4	0	20.3
FN	—	—	4.2	4.2	—	—	5.4	5.4

Abbreviations: FN, febrile neutropenia; G, grade; ISR, infusion site reaction.

## 4 DISCUSSION

This study examined the administration of palonosetron or granisetron combined with dexamethasone and Fos for preventing CINV in breast cancer patients who placed on AC‐based regimen. This is the first randomized study to report the efficacy of Fos for patients with breast cancer receiving AC‐based regimen. Palonosetron was not found to be better than granisetron in terms of the CR during the delayed phase, acute phase, and the overall phase, although some secondary endpoints, including delayed nausea and patient‐reported frequency of emesis, were improved in patients who were administered palonosetron. Preplanned subgroup analysis revealed a history of motion sickness as a distinct risk factor; however, its presence or absence did not help predict the treatment effects of palonosetron.

A phase 3 trial (the TRIPLE study) compared the treatment effects of palonosetron and granisetron in combination with aprepitant and dexamethasone in patients receiving CDDP and showed that palonosetron is better than granisetron in terms of the CR during the delayed phase (67.2% vs 59.1%; *P* = .0142).^15^ Interestingly, the CR during the delayed phase in the granisetron regimen was similar in both TRIPLE (59.1%) and WJOG 6811B (60.4%) studies, whereas the CR during delayed phase in the palonosetron regimen was slightly different (67.2% in TRIPLE vs 62.3% in WJOG 6811B). Two reasons may explain the difference in the treatment effect of palonosetron between the TRIPLE and WJOG 6811B studies. First is the difference in the chemotherapy drug: CDDP in the TRIPLE study vs AC‐based regimen in the WJOG 6811B study. The CINV peak in AC‐based regimen tends to be later compared with the CDDP regimen,^16^ although in the PROTECT study, palonosetron improved CR during the delayed phase (from 50% to 61.1%) in AC/EC group, which was similar in the CDDP group (from 40.6% to 53.5%). Second is the patient background: 75% of patients in the TRIPLE study were males, whereas all patients in the WJOG 6811B study were females. Tamura et al conducted a nationwide registry study in Japan and concluded that sex remains a distinct risk factor for CINV, even among patients receiving NK‐1 RAs.^17^ The reason why palonosetron did not work as desired (combined palonosetron with 3‐days dexemathasone and NK1‐RA after AC‐based regimen) needs further study.

Navari et al reported the usefulness of olanzapine as the fourth drug with dexamethasone, 5‐HT‐3 RA, and NK‐1 RA. They found that olanzapine improves the CR during the delayed phase from 52% to 67% and no nausea during the delayed phase from 25.4% to 42.4%.^18^ The newest ASCO guideline recommends upfront olanzapine for all patients with breast cancer on the basis of this study,^19^ although MASCC/ESMO guidelines stated that olanzapine may be considered with a triple regimen, particularly when nausea is an issue.

Another issue related to safety was ISR, observed in one fifth of the patients in both arms of the study. This frequency is higher than the data (2.7%) reported in the EASE study.^7^ A recent study reported Fos administered in the peripheral vein with AC‐based regimen would lead to a higher risk of ISR.^20^ This study reproduced a similar result. The timing of ISRs onset may vary because half of the physicians reported ISRs to be related to Fos, whereas most of them also reported ISRs to be related to chemotherapy. Even if ISRs begin during chemotherapy infusion, the high frequency of ISR in this study strongly suggests Fos increases the risk. We do believe that a follow‐up study using Fos with AC‐based regimen should avoid dosing in the peripheral veins.

This study, however, has several limitations. First, dexamethasone was administered on days 1, 2, and 3, although the newest guidelines recommend single dose dexamethasone for patients receiving AC‐based regimen. This is also supported by a recent study^21^ that proved noninferiority of single dose dexamethasone as triplet regimen with palonosetron and aprepitant for patients receiving HEC (including AC‐based regimen). Omitting dexamethasone on day 2 and day 3 might affect the conclusion of this study. Second, the sample size was relatively small for a phase 3 study. However, the difference in the CR during the delayed phase was small (1.9%) and the CR during the overall phase was identical in regimens A and B; hence, a larger study might not necessarily lead to a clinically meaningful difference.

## 5 CONCLUSION

Palonosetron exerts efficacy against delayed CINV which is not better than that of the combination with granisetron for patients with breast cancer receiving 3 days dexamethasone and Fos after AC‐based regimen, even though palonosetron reduced significantly delayed nausea. Both palonosetron and granisetron combined with steroids and NK‐1 RAs are good options for CINV prevention in patients with breast cancer receiving AC‐based regimen. These patients are still at a higher risk for nausea, especially during the delayed phase. Administration of Fos in the peripheral vein with AC‐based regimen should be avoided because it leads to higher risk of ISR.

## CONFLICT OF INTEREST

Matsumoto reports grants and personal fees from ONO, grants from MSD, grants from ICON, personal fees from Kyowa Hakko Kirin, personal fees from Chugai, grants and personal fees from AstraZeneca, personal fees from Taiho, grants and personal fees from Novartis, personal fees from Eisai, personal fees from Nihon Kayaku, outside the submitted work; TAKAHASHI reports personal fees from Astra Zeneca, personal fees from Eisai, personal fees from pfizer, personal fees from Eli Lilly, personal fees from Kyowa Kirin, personal fees from Taiho, personal fees from Nipponkayaku, personal fees from Chugai, outside the submitted work; Osaki reports grants and personal fees from AstraZeneca KK, grants from Eisai Co., Ltd., grants from MSD KK, grants from Ono Pharmaceutical Co., Ltd., grants and personal fees from Kyowa Hakko Kirin Co., Ltd., grants and personal fees from Daiichi Sankyo Co., Ltd., grants from Taiho Pharmaceutical Co., Ltd., grants from Sawai Pharmaceutical Co., Ltd., grants and personal fees from Chugai Pharmaceutical Co., Ltd., grants from Nippon Kayaku Co., Ltd., grants from Nihon Medi‐Physics Co., Ltd., grants and personal fees from Novartis Pharma KK, grants from Hamamatsu Photonics KK, grants from Parexel International Inc, grants from Fuji Pharma Co., outside the submitted work; Takano reports grants and personal fees from Daiichi‐Sankyo, grants and personal fees from Kyowa Hakko Kirin, grants and personal fees from Eisai, personal fees from Pfizer, grants from Ono, grants from MSD, grants from Merck Serono, grants from Taiho, grants from Novartis, grants from Chugai, outside the submitted work; Naito reports other from Pfizer, other from Taiho, other from Nippon Kayaku, other from Eli Lilly, other from AstraZeneca, other from Merck Serono, other from Bayer, other from Meiji Seika, other from Roche Diagnostics, other from Novartis, other from Taiho, other from Chugai Pharmaceutical, other from Eisai, outside the submitted work; K.Aogi received personal fees as honoraria from Chugai Pharmaceutical, Eisai, Sanofi, SRL, AstraZeneca, Taiho Pharmaceutical, Novartis Pharma, Daiichi Sankyo, Mochida Pharmaceutical, Ono Pharmaceutical, Otsuka Pharmaceutical, and Eli Lilly Japan, and his institution received research funds from Chugai Pharmaceutical, Eisai and Sanofi; Tokunaga reports personal fees from Eisai Co, Ltd, personal fees from Chugai Pharmaceutical Co, Ltd, personal fees from Kyowa Hakko Kirin Co., Ltd., personal fees from Novartis Pharma Co, Ltd, outside the submitted work; Imamura reports personal fees from Chugai Pharmaceutical Co., Ltd., personal fees from Ono Pharmaceutical Co., Ltd., personal fees from Taiho Pharmaceutical Co., Ltd., personal fees from MSD KK, personal fees from AstraZeneca KK, personal fees from Nippon Boehringer Ingelheim Co., Ltd., personal fees from Nippon Chemiphar Co., Ltd., outside the submitted work;. Imoto reports grants from Chugai and Taiho, personal fee from AstraZeneca, outside of the submitted work; Saeki reports grants and personal fees from AstraZeneca KK, grants from Eisai Co., Ltd., grants from MSD KK, grants and personal fees from Ono Pharmaceutical Co., Ltd., grants and personal fees from Kyowa Hakko Kirin Co., Ltd., grants from Daiichi Sankyo Co., Ltd., grants and personal fees from Taiho Pharmaceutical Co., Ltd., grants from Sawai Pharmaceutical Co., Ltd., grants and personal fees from Chugai Pharmaceutical Co., Ltd., grants from Nippon Kayaku Co., Ltd., grants from Nihon Medi‐Physics Co., Ltd., grants and personal fees from Novartis Pharma KK, grants from Hamamatsu Photonics KK, grants from Parexel International Inc, grants and personal fees from Fuji Pharma Co., outside the submitted work. The other authors made no disclosures.

## AUTHOR CONTRIBUTIONS

Koji Matsumoto was involved in conceptualization, data curation, formal analysis, funding acquisition, investigation, methodology, project administration, resources, supervision, validation, visualization, and writing (original draft, review, and editing). Masato Takahashi was involved in investigation, resources, and writing‐review and editing. Kazuhiko Sato, Akihiko Oosaki, Toshimi Takano, Yoichi Naito, Kazuo Matsuura, Kenjiro Aogi, Kimiko Fujiwara, Kenji Tamura, Motoi Baba, Shinya Tokunaga, Gen Hirano, Shigeru Imoto, Chieko Miyazaki, Kazuhiro Yanagihara, and Toshiaki Saeki were involved in investigation, resources, and writing‐review and editing.. Chiyo K. Imamura was involved in project administration, resources, and writing‐review and editing. Yasuchika Chiba was involved in data curation, formal analysis, methodology, validation, and writing‐review and editing.

## Data Availability

The data that support the findings of this study are available from WJOG. Restrictions apply to the availability of these data, which were used under license for this study. Data are available from the authors with the permission of WJOG.

## References

[1] Hesketh PJ , Bohlke K , Lyman GH , et al. Antiemetics: American Society of Clinical Oncology focused guideline update. J Clin Oncol. 2016;34:381‐386. 26527784 10.1200/JCO.2015.64.3635

[2] Network NCC . NCCN Cliincal Practice Guidlines in Oncology Antiemesis.2012.

[3] Roila F , Molassiotis A , Herrstedt J , et al. 2016 MASCC and ESMO guideline update for the prevention of chemotherapy‐ and radiotherapy‐induced nausea and vomiting and of nausea and vomiting in advanced cancer patients. Ann Oncol. 2016;27(suppl 5):v119‐v133. 27664248 10.1093/annonc/mdw270

[4] Saito M , Aogi K , Sekine I , et al. Palonosetron plus dexamethasone versus granisetron plus dexamethasone for prevention of nausea and vomiting during chemotherapy: a double‐blind, double‐dummy, randomised, comparative phase III trial. Lancet Oncol. 2009;10:115‐124. 19135415 10.1016/S1470-2045(08)70313-9

[5] Likun Z , Xiang J , Yi B , Xin D , Tao ZL . A systematic review and meta‐analysis of intravenous palonosetron in the prevention of chemotherapy‐induced nausea and vomiting in adults. Oncologist. 2011;16:207‐216. 21282670 10.1634/theoncologist.2010-0198 PMC3228089

[6] Basch E , Prestrud AA , Hesketh PJ , et al. Antiemetics: American Society of Clinical Oncology clinical practice guideline update. J Clin Oncol. 2011;29:4189‐4198. 21947834 10.1200/JCO.2010.34.4614 PMC4876353

[7] Grunberg S , Chua D , Maru A , et al. Single‐dose fosaprepitant for the prevention of chemotherapy‐induced nausea and vomiting associated with cisplatin therapy: randomized, double‐blind study protocol–EASE. J Clin Oncol. 2011;29:1495‐1501. 21383291 10.1200/JCO.2010.31.7859

[8] Maemondo M , Masuda N , Sekine I , et al. A phase II study of palonosetron combined with dexamethasone to prevent nausea and vomiting induced by highly emetogenic chemotherapy. Ann Oncol. 2009;20:1860‐1866. 19561037 10.1093/annonc/mdp195

[9] Segawa Y , Aogi K , Inoue K , et al. A phase II dose‐ranging study of palonosetron in Japanese patients receiving moderately emetogenic chemotherapy, including anthracycline and cyclophosphamide‐based chemotherapy. Ann Oncol. 2009;20:1874‐1880. 19605507 10.1093/annonc/mdp243

[10] Gralla R , Lichinitser M , Van der Vegt S , et al. Palonosetron improves prevention of chemotherapy‐induced nausea and vomiting following moderately emetogenic chemotherapy: results of a double‐blind randomized phase III trial comparing single doses of palonosetron with ondansetron. Ann Oncol. 2003;14:1570‐1577. 14504060 10.1093/annonc/mdg417

[11] Aapro MS , Grunberg SM , Manikhas GM , et al. A phase III, double‐blind, randomized trial of palonosetron compared with ondansetron in preventing chemotherapy‐induced nausea and vomiting following highly emetogenic chemotherapy. Ann Oncol. 2006;17:1441‐1449. 16766588 10.1093/annonc/mdl137

[12] Eisenberg P , Figueroa‐Vadillo J , Zamora R , et al. Improved prevention of moderately emetogenic chemotherapy‐induced nausea and vomiting with palonosetron, a pharmacologically novel 5‐HT3 receptor antagonist: results of a phase III, single‐dose trial versus dolasetron. Cancer. 2003;98:2473‐2482. 14635083 10.1002/cncr.11817

[13] Warr DG , Hesketh PJ , Gralla RJ , et al. Efficacy and tolerability of aprepitant for the prevention of chemotherapy‐induced nausea and vomiting in patients with breast cancer after moderately emetogenic chemotherapy. J Clin Oncol. 2005;23:2822‐2830. 15837996 10.1200/JCO.2005.09.050

[14] Takahashi T , Hoshi E , Takagi M , Katsumata N , Kawahara M , Eguchi K . Multicenter, phase II, placebo‐controlled, double‐blind, randomized study of aprepitant in Japanese patients receiving high‐dose cisplatin. Cancer Sci. 2010;101:2455‐2461. 20718754 10.1111/j.1349-7006.2010.01689.x PMC11159452

[15] Suzuki K , Yamanaka T , Hashimoto H , et al. Randomized, double‐blind, phase III trial of palonosetron versus granisetron in the triplet regimen for preventing chemotherapy‐induced nausea and vomiting after highly emetogenic chemotherapy: TRIPLE study. Ann Oncol. 2016;27:1601‐1606. 27358385 10.1093/annonc/mdw220

[16] Hesketh PJ , Warr DG , Street JC , Carides AD . Differential time course of action of 5‐HT3 and NK1 receptor antagonists when used with highly and moderately emetogenic chemotherapy (HEC and MEC). Support Care Cancer. 2011;19:1297‐1302. 20623144 10.1007/s00520-010-0944-4

[17] Tamura K , Aiba K , Saeki T , et al. Testing the effectiveness of antiemetic guidelines: results of a prospective registry by the CINV Study Group of Japan. Int J Clin Oncol. 2015;20:855‐865. 25681876 10.1007/s10147-015-0786-7

[18] Navari RM , Qin R , Ruddy KJ , et al. Olanzapine for the prevention of chemotherapy‐induced nausea and vomiting. N Engl J Med. 2016;375:134‐142. 27410922 10.1056/NEJMoa1515725 PMC5344450

[19] Hesketh PJ , Kris MG , Basch E , et al. Antiemetics: American Society of clinical oncology clinical practice guideline update. J Clin Oncol. 2017;35:3240‐3261. 28759346 10.1200/JCO.2017.74.4789

[20] Leal AD , Kadakia KC , Looker S , et al. Fosaprepitant‐induced phlebitis: a focus on patients receiving doxorubicin/cyclophosphamide therapy. Support Care Cancer. 2014;22:1313‐1317. 24402411 10.1007/s00520-013-2089-8 PMC3969765

[21] Ito Y , Tsuda T , Minatogawa H , et al. Placebo‐controlled, double‐blinded phase III study comparing dexamethasone on day 1 with dexamethasone on days 1 to 3 with combined neurokinin‐1 receptor antagonist and palonosetron in high‐emetogenic chemotherapy. J Clin Oncol. 2018;36:1000‐1006. 29443652 10.1200/JCO.2017.74.4375

